# Regulating the regulatory T cells as cell therapies in autoimmunity and cancer

**DOI:** 10.3389/fmed.2023.1244298

**Published:** 2023-09-27

**Authors:** Hamed Hosseinalizadeh, Fatemeh Rabiee, Negar Eghbalifard, Hamid Rajabi, Daniel J. Klionsky, Aryan Rezaee

**Affiliations:** ^1^Department of Medical Biotechnology, Faculty of Paramedicine, Guilan University of Medical Sciences, Rasht, Iran; ^2^Department of Pharmacology and Pharmaceutical Sciences Research Center, Isfahan University of Medical Sciences, Isfahan, Iran; ^3^Faculty of Medicine, Isfahan University of Medical Sciences, Isfahan, Iran; ^4^Faculty of Medicine, ShahreKord University of Medical Sciences, Shahrekord, Iran; ^5^Department of Molecular, Cellular and Developmental Biology, Life Sciences Institute, University of Michigan, Ann Arbor, MI, United States; ^6^Student Research Committee, School of Medicine, Iran University of Medical Sciences, Tehran, Iran

**Keywords:** autoimmunity, cancer, immunotherapy, reprogramming, Treg

## Abstract

Regulatory T cells (Tregs), possess a pivotal function in the maintenance of immune homeostasis. The dysregulated activity of Tregs has been associated with the onset of autoimmune diseases and cancer. Hence, Tregs are promising targets for interventions aimed at steering the immune response toward the desired path, either by augmenting the immune system to eliminate infected and cancerous cells or by dampening it to curtail the damage to self-tissues in autoimmune disorders. The activation of Tregs has been observed to have a potent immunosuppressive effect against T cells that respond to self-antigens, thus safeguarding our body against autoimmunity. Therefore, promoting Treg cell stability presents a promising strategy for preventing or managing chronic inflammation that results from various autoimmune diseases. On the other hand, Tregs have been found to be overactivated in several forms of cancer, and their role as immune response regulators with immunosuppressive properties poses a significant impediment to the successful implementation of cancer immunotherapy. However, the targeting of Tregs in a systemic manner may lead to the onset of severe inflammation and autoimmune toxicity. It is imperative to develop more selective methods for targeting the function of Tregs in tumors. In this review, our objective is to elucidate the function of Tregs in tumors and autoimmunity while also delving into numerous therapeutic strategies for reprogramming their function. Our focus is on reprogramming Tregs in a highly activated phenotype driven by the activation of key surface receptors and metabolic reprogramming. Furthermore, we examine Treg-based therapies in autoimmunity, with a specific emphasis on Chimeric Antigen Receptor (CAR)-Treg therapy and T-cell receptor (TCR)-Treg therapy. Finally, we discuss key challenges and the future steps in reprogramming Tregs that could lead to the development of novel and effective cancer immunotherapies.

## 1. Introduction

Immunotherapy seeks to utilize the immune system in the management of a diverse range of illnesses (1–4). Nonetheless, in order for a particular therapy to be efficacious, it is essential that it precisely target an immune response against the illness while simultaneously safeguarding the host from autoimmunity and producing the desired antitumor outcomes (5–7). Such an effect is scrutinized especially in the context of cancer and autoimmune disorders (8). In the tumor microenvironment, Tregs (CD4+ FOXP3+ regulatory T cells), formerly known as suppressor T cells, promote cancer immune evasion through the suppression of antitumor T effector responses (9, 10). As a result, they represent the primary obstacles to cancer immunotherapy. On the other hand, a deficiency in Treg function fosters T effector responses against self-antigens, which can ultimately lead to autoimmune disease (8, 11–13). Therefore, the disruption of the homeostatic balance between T effector and Treg cells is frequently associated with both cancer and autoimmunity (14–16).

The importance of Tregs in cancer and autoimmune diseases has made them a potentially critical target for these illnesses (17, 18). It is increasingly evident that the development of effective therapeutic strategies to reprogram Treg functionality holds great promise for immunotherapy, both in inducing tolerance in autoimmune diseases and advancing immune-based treatments for cancer (19). In cancer, the selective reprogramming of Treg cells through molecular modules serves not only to reduce their immunosuppressive activity but also may have the potential to enhance the efficacy of cancer treatment by converting immunosuppressive Tregs into cancer-specific immunostimulatory cells (19, 20).

This review provides comprehensive data, focusing on novel drugs and strategies aimed at reprogramming Tregs for the treatment of cancer and autoimmunity. The section entitled “Treg-based therapies in cancer” emphasizes two critical aspects of TI(tumor-infiltrating)-Tregs that, when perturbed, can modify their function: (i) their highly activated phenotype *via* stimulatory cell surface receptors; and (ii) their metabolic status. In the section “Treg-based therapies in autoimmunity,” we discuss two therapies that are currently being investigated for the cure and prevention of autoimmune diseases: (i) autoantigen-specific TCR Tregs and (ii) autoantigen-specific CAR Tregs. These therapies suppress effector T cell function and promote Tregs, restoring immune homeostasis and tolerance by promoting and activating Tregs. Finally, we review the future steps of Treg reprogramming to highlight the potential of Treg reprogramming in the treatment of both cancer and autoimmunity.

## 2. Role of Tregs in tumor

For decades, researchers have investigated the association between Tregs and tumor microenvironments. Tregs are present in various cancers’ tumor microenvironment (TME), including melanoma, lung, ovarian, pancreatic, breast, and stomach cancers, and high Treg infiltration has been linked to low survival rates and a poor prognosis (21, 22). Tregs can be generated and differentiated by conventional T cells in the TME, and these cells have a potent immunosuppressive effect that impedes antitumor immunity and promotes tumor growth. Tregs are crucial in the tumor’s immune escape and can repress immune effector cell activity in diverse forms in tumor-bearing hosts. Therefore, Tregs function as a double-edged sword, with a protective role in maintaining immune homeostasis and a pathological role in the inhibition of effector cells in TME (23). Tregs hinder immune responses against tumors by a variety of mechanisms. Tregs can release immunosuppressive molecules that can suppress antitumor immunity, such as TGF-β (Transforming growth factor-β), IL-10, and IL-35. TGF-β can inhibit T-helper (Th) 1, NK, and CTLs(Cytotoxic T lymphocytes) cell responses and convert NK cells in the TME to innate type 1 lymphoid cells (24). Moreover, TGF-β signaling can stimulate the differentiation of suboptimally stimulated peripheral CD4+ T cells into Tregs which then promotes the development of immune tolerance and immunosuppression in the TME (24, 25). It should be noted that both immune and nonimmune cells can generate TGF-β, and the role of TGF-β derived from Treg cells remains controversial (26, 27). TGF-β and IL10 induce the conversion of resident fibroblasts into cancer-associated fibroblasts (CAF) and monocytes into tumor-associated macrophages (TAM), respectively (28, 29). CAFs and TAMs are capable of inducing effector T cell apoptosis, thereby facilitating tumor evasion from antitumor immunity (30). The production of IL-10 by Tregs may be crucial for the survival and proliferation of tumor cells, as it regulates antitumor immunity. Its expression has been associated with an unfavorable prognosis in various cancers (31). The effects of IL-10 on the TME are varied, and IL-35 can reinforce its immunosuppressive impact, which is also generated by Tregs (31). Tregs producing IL-35 accumulated in the TME and impeded effector T cell activation and their effector function by inducing the expression of several inhibitory receptors such as PD-1 (Programmed cell death protein 1), Tim-3(T cell immunoglobulin and mucin-3)and BLIMP1(B-lymphocyte-induced maturation protein-1)-dependent exhaustion (32).

It is noteworthy that IL-35 and IL-10 have distinct immunomodulatory functions. Tregs producing IL-35 promote effector T cell exhaustion, while Tregs producing IL-10 inhibit the cytotoxic effector function of effector T cells. To some extent, Treg cells can also activate major pathways that regulate apoptosis, such as the FAS-FasL and TNF-TNFR-mediated signaling in response to specific antigens (33). Tregs can prompt tumors to become resistant to immune checkpoint inhibitors (ICIs). While the precise processes through which Tregs can promote tumor resistance to ICIs are not fully comprehended, several molecules and signaling pathways, such as Phosphatidylinositol 3-kinase (PI3K), have been associated with the resistance of Tregs to approved ICIs (23). Other roles played by Tregs include the immediate destruction of other cells by secreting granzyme and perforin, and the production and release of cAMP to disrupt the metabolism of other cells.

## 3. Role of Tregs in autoimmunity

Tregs play a pivotal role in preventing a multitude of autoimmune diseases by virtue of their capacity to maintain self-tolerance. Hence, the malfunctioning, paucity of Tregs, and the resistance of effector cells toward Treg immunoregulatory mechanisms can initiate autoimmune diseases. This is in line with earlier research, which indicates that several human autoimmune diseases exhibit abnormalities in Treg function or peripheral Treg cell counts (34). The mechanism by which Tregs inhibit autoreactivity is that when activated *via* their TCR and the costimulatory molecule CD28, Tregs secrete anti-inflammatory cytokines such as TGF-β, IL10, and IL35 that target different cell types at the site of inflammation (35). These anti-inflammatory cytokines induce the production of tolerogenic APCs, which promote the anergy of autoreactive memory effector CD4+ T cells that bind to their MHC molecules (36). Tregs have also been found to suppress autoreactive effector T cells *via* IL-2 consumption or the induction of cell death through granzyme and perforin. Tolerogenic APCs can also induce the expression of PD-L1 (CD274 molecule), serving as a pivotal mechanism for the induction of CD8+ T cell exhaustion following autoimmune activation (37). The expression of FOXP3 is believed to be modified or reduced in various diseases, including type 1 diabetes (T1D) and multiple sclerosis (MS) in humans, as well as the Scurfy phenotype (which involves the complete absence of T cell regulatory function) in mice (38–40). Apart from the FOXP3 expression loss, which serves as the main regulator of Treg cells, a specific epigenetic signature is a distinguishing feature of Treg cells (41). Both components(epigenetic and FOXP3 expression) are fundamental in the preservation of Treg cell function, and it is probable that the perturbation of FOXP3 expression or epigenetic modifications may result in Treg cell instability and aberrant plasticity, which are commonly observed in a variety of autoimmune diseases. Moreover, there is compelling evidence showing that the impairment of Treg function can be linked to a decline in thymic production and a decrease in the mutual interplay between CD58 and CD2, both of which belong to the immunoglobulin family (42). In EAE (experimental autoimmune encephalomyelitis), it has been observed that the transmission of Tregs was sufficient in mitigating the severity of EAE (43). Conversely, the removal of Tregs has been shown to exacerbate the condition in EAE models (43). Despite recent advancements in comprehending Treg functions in pathological circumstances, more research is required to explicate the mechanisms that underlie Treg cell instability and plasticity. This will enable the modulation of Treg cell function in autoimmune pathologies and other diseases like cancer.

## 4. Treg-based therapies in cancer

The recent success of various immune-based therapies for cancer that activate cytotoxic T cells to attack cancer cells has brought about a revolutionary change in cancer treatment. However, a significant number of patients do not respond favorably to these immune-stimulating therapies, highlighting the requirement for therapies that enhance the immune response beyond a simple boost. Currently, the major challenge hindering the success of tumor immunotherapy is overcoming immunosuppressive TME (20). Tregs are present in almost all cancers and serve as immunosuppressive regulators of immune responses, representing a significant obstacle to the success of cancer immunotherapy (44, 45). The requirement for a novel approach directed toward the targeted alteration of intratumoral regulatory T cells has been expedited due to the fact that general inhibition of regulatory T cells results in significant autoimmune toxicity (45). Hence, the specific reprogramming of Treg cells may decrease the frequency of immune-related unfavorable events frequently linked with systemic approaches intended to eradicate Treg cells, which may result in the discontinuation of therapy and subsequent loss of therapeutic benefit (46). Reprogramming Tregs has the potential to achieve two significant benefits in cancer therapy. First, it can evade immune regulation in tumors by eliminating immunosuppressive cells. Second, it can enhance the effectiveness of cancer treatment by transforming immunosuppressive Tregs into immunostimulatory cells, specifically within cancer (33). To reprogram the TI (tumor infiltrating)-Treg function, it is necessary to focus on targeting the unique properties of TI-Tregs, which include their activation condition *via* stimulatory cell surface receptors, metabolic condition, and transcriptional condition as influenced by critical transcription factors and chromatin regulators (20, 47).

### 4.1. Reprogramming of Tregs in highly activated phenotype

TI-Tregs exhibit highly activated phenotype. A number of receptors, including CTLA4 (cytotoxic T-lymphocyte associated protein 4), GITR (TNF receptor superfamily member 18), CD25, CD28, CD39, OX40, PD1 (programmed cell death 1), LAG3 (lymphocyte activating 3), NRP1 (neuropilin 1), chemokine receptors, and NT5E are constitutively expressed in human Tregs and become visible on the cell surface upon activation in the TME. These receptors have the crucial role in regulating the immune response against tumors (48–50). Therefore, targeting Tregs in tumors and reprogramming their functions to the highly activated state of TI-regs or their particular differentiation state is an effective approach.

#### 4.1.1. OX40

The surface receptor OX40 is expressed by both effector T cells and Tregs. Despite its role as a potent costimulatory molecule for activated effector T cells, OX40 plays a critical role in regulating FOXP3 Tregs. According to Vue et al., activation of OX40 on mature FOXP3 Tregs completely disables their ability to inhibit effector T cell proliferation and cytokine production. Interestingly, OX40 signaling on Tregs does not appear to affect their survival and proliferation but suppresses FOXP3 expression (51). OX40 consistently impedes the capacity of TGFB to elicit FOXP3 expression in activated effector T cells. Consequently, OX40 functions as a potent negative regulator of both inherent FOXP3 Tregs and Tregs that are produced by activated effector T cells. This illustrates that OX40 governs a pivotal checkpoint in Treg homeostasis (51). The co-stimulation of OX40 has emerged as a promising method to combat the suppressive effects of Tregs (52). OX40 agonists have been observed to augment antitumor immunity in multiple tumor models and are currently being investigated as a new cancer immunotherapy target in clinical trials. The primary OX40 agonist antibodies employed in clinical research are MEDI0562, MEDI6469, INBRX-106, PF04518600, and SL279252 (53). Another novel approach for improving antitumor immunity involves the *in situ* reprogramming of tumor cells into “artificial” antigen-presenting cells (APCs) that express OX40L using nanoparticles that are loaded with an OX40L plasmid. This method has demonstrated the capacity to induce T cell proliferation *in vitro*, while also eliciting potent antitumor immune responses *in vivo* without any notable toxicity (54). While targeted therapy for OX40 has exhibited remarkable outcomes in mice with tumors, preliminary clinical evidence suggests that its efficacy in humans is moderate when utilized independently. Nevertheless, when employed with immunotherapies that target inhibitory receptors such as anti-PD-1 and anti-PD-L1, OX40 costimulation demonstrates potential as a promising strategy (55).

#### 4.1.2. CD28

The secondary stimulus for Treg function is activated concurrently with TCR stimulation and facilitated by the costimulatory receptor CD28. The protein CD28 from the immunoglobulin superfamily contains a single “V-like” extracellular domain that positively regulates Treg cell response. It is the most typical and best- studied costimulatory transmembrane protein (56). CD28 is of critical importance in the development of Treg cells in the thymus, as well as in the survival, proliferation, suppressor function, and homeostasis of Treg cells in the periphery (57, 58). However, it is still unknown how CD28 regulates Treg development and homeostasis. In addition, CD28 plays a crucial role in preventing T cell anergy by providing a co-stimulatory signal. Without this signal, T cells may either remain inactive or develop tolerance to antigens. Therefore, CD28 is identified as an essential second signal for T cell activation. In the nonobese diabetic (NOD) mouse model, wherein CD28 or its ligands (CD80 [B7-1] and CD86 [B7-2]) have been knocked out, the number of natural Tregs (nTregs) decreased significantly, resulting in accelerated autoimmunity in mice (58). The development of CD28 blockers could potentially offer a more targeted therapy for cancer. Drugs like abatacept (CTLA4-Ig) and belatacept (LEA29Y) affect the CD28 signaling pathway by acting as blocking agents for CD28/CD80-CD86 interactions. Abatacept, for example, inhibits CD28 from binding to CD80-CD86 on the APC surface, thereby hindering CD28-mediated costimulatory signaling that is essential for Treg cell activation and reducing the resulting downstream inflammation response (59). These medications are employed in clinical settings to manage autoimmune diseases like psoriasis and rheumatoid arthritis (RA), wherein genetic abnormalities or modified posttranslational variations of the CTLA-4 gene or its promoter are detected. However, more research is needed to determine how strong the immunosuppressive effects of these drugs are in cancer settings. Inhibition of downstream components of the CD28 signaling pathway is another potential target for modulating Treg cell activation. Vang et al. demonstrated that the effective development of Tregs requires the P187YAP motif in the cytoplasmic tail of CD28 (60). The CBM complex (CARD11/CARMA1-BCL10-MALT1) is activated by the LCK-PRKC (protein kinase C) pathway, which is linked to CD28 through this motif (61). This complex then activates the JNK2 complex and the IKK complex, which, in turn, triggers the activation of the classical NF-κB pathway (60, 61). REL is one of the NF-κB family members required for the development of progenitor Tregs or the development of mature Tregs. A xanthine derivative called PTXF (pentoxifylline) has specific and dose-dependent effects on REL and exhibits competitive and nonselective phosphodiesterase inhibitory activity (62). Ghosh et al. demonstrated that in a mouse model of melanoma inhibition of C-Rel by PTXF, similar to genetic deletion of C-Rel, reduced Tregs and improved antitumor response (63). Inhibition of MALT1 in tumor-bearing mice with a drug induces Treg cells to secrete the immunostimulatory cytokine IFNγ only in tumor tissue, resulting in stunted tumor growth. This antitumor effect is due to macrophage activation and upregulation of MHC class I on tumor cells (64). EZH2 (enhancer of zeste 2 polycomb repressive complex 2 subunit) is expressed in Treg cells *via* CD28 signaling (1). Once activated, FOXP3 and EZH2 can develop a complex crucial for preserving the identity of nTregs. Suppression of EZH2 in Tregs (using *Foxp3*-cre *ezh2^fl/fl^* mice) and employing CPI-1205 (a pharmacological inhibitor of EZH2) resulted in functional modifications in Tregs and improved cytotoxic activity of effector T cells (65).

#### 4.1.3. CTLA4

CTLA4 is a related inhibitor of the costimulatory T-cell molecule CD28. While CD28 promotes T-cell activation, CTLA4 serves as an immune checkpoint and downregulated T-cell responses (66). Similar to CD28, CTLA4 binds to CD80 and CD86 on APCs, but with higher affinity and avidity than CD28, allowing it to outcompete CD28 in terms of ligands. Activation of CD28 induces expression of CTLA4 as a negative feedback mechanism in effector T cells and inhibits activation of effector T cells, whereas expression of CTLA4 in TI-Tregs, most likely due to their highly activated state, enhances their immunosuppressive activity in the TME (67). Monoclonal antibodies directed toward CTLA-4 have been found to provide an immunotherapeutic effect against cancer (CITE), however, they are also associated with inducing severe immunotherapy-related adverse events (irAE). Despite this, the targeting of CTLA-4 remains a valuable approach for cancer immunotherapy owing to its remarkable long-term benefits provided the irAEs could be effectively managed (68). Further research is warranted to design safer reagents targeting CTLA-4. Several therapeutic molecules are currently being developed to selectively knock down or disrupt CTLA4 function in TI-Tregs. Drugs that block CTLA4 are called ICIs (immune checkpoint inhibitors) (66, 69). These drugs include ipilimumab (Yervoy) and tremelimumab. Inhibition of CTLA4 by ipilimumab and tremelimumab allows for CD28-mediated positive signaling and activation of cytotoxic T-cell responses (69). Ipilimumab was the first antibody approved by the FDA. It is a fully human anti-CTLA4 (IgG1) monoclonal antibody that has been shown to improve the long-term survival of melanoma patients (70).

#### 4.1.4. GITR

GITR (TNF receptor superfamily member 18) is categorized under the TNFR superfamily, and its expression is more common in Treg cells as compared to conventional CD4 and CD8 T cells. GITR can interact with its corresponding ligand, GITRL, which is expressed in APCs and endothelial cells (71, 72). The activation of GITR on Treg cells leads to instability and reduction of suppressive function, whereas activation of GITR in conventional T cells results in a co-stimulatory signal that enhances T effector cell viability, cytokine production, and effector function (72). Studies have provided evidence that the resistance to immunotherapy in glioblastoma mice models can be effectively reduced through the targeted activation of Treg cells by GITR. In a study conducted by Amoozgar et al., it was demonstrated that the activation of GITR in Treg cells by an agonistic antibody (αGITR) led to the transformation of immunosuppressive Treg cells in the TME into immunostimulatory Th1-like CD4 T cells, enhancing the immune response against the tumor (73). ΑGITR promotes the differentiation of CD4 Treg cells into CD4 effector T cells, reduced the ability of Treg cells to suppress effector T cells, and induced potent antitumor effector cells in GBM (73). Other potent GITR mAbs currently in ongoing clinical trials include MEDI1873 (NCT02583165) (74), AMG 228 (NCT02437916) (75), BMS-986156 (NCT02598960) (76), and GWN323 (NCT02740270) (77). A Phase 1 clinical trial administered the anti-GITR antibody TRX518 to patients with cancer that cannot be cured. The study established that TRX518 is safe to use as monotherapy and has immune effects (78). However, although Tregs were reduced and the ratios of T effector cells to Tregs increased, there were no notable clinical responses. Similarly, in mice with advanced tumors, GITR agonist alone could not activate effector T cells because of persistent exhaustion (78, 79). Overcoming the resistance of advanced tumors to anti-GITR monotherapy may be achieved through T-cell reinvigoration with other immune checkpoint inhibitors, such as PD-1 blockade. These findings have prompted the investigation of TRX518 in combination with the PD-1 pathway blockade in patients with advanced refractory tumors (NCT02628574) (78).

#### 4.1.5. CD25

The IL2RA (interleukin-2 receptor subunit alpha)/CD25 is expressed on activated CD4+ and CD8+ T cells, as well as on Tregs, in a constitutive manner. It is noteworthy that Tregs exhibit a greater upregulation of IL2RA expression when compared to CD4+ and CD8+ T cells. This observation implies that targeting IL2RA could be a promising strategy for cancer therapy, as supported by prior research (80). The development, control, proliferation, and maintenance of Tregs depend on IL2. The binding of IL2 to IL2R leads to phosphorylation, dimerization and translocation of STAT5 (signal transducer and activator of transcription 5) to the nucleus, where they direct the transcription of target genes required for the Treg immunosuppressive phenotype, largely through direct regulation of FOXP3 expression (81). CD25 also interferes with the optimal functioning of effector T cells by depriving surrounding effector T cells of IL2. IL2 is necessary for the differentiation and fate of effector T cells after immune activation (82). Therefore, an efficient strategy to reprogram Tregs would entail the creation of a recombinant IL-2 that demonstrates heightened selectivity toward the IL-2 receptor on natural killer (NK) and naïve CD8 T-cells, while abstaining from binding to the IL-2 receptor on Tregs. The first approach targeting IL2RA was the use of denileukin diptitox, a fusion protein of IL2 and diphtheria toxin (83). More recently, the recombinant anti-IL2RA immunotoxins and daclizumab have been evaluated for their ability to block IL2 binding to IL2RA in Tregs (83). However, the mechanism of action of these drugs remains to be elucidated. Proleukin (recombinant human interleukin-2) has been identified as an effective therapy for metastatic melanoma and renal cell carcinoma (RCC) (84–86). However, the need for the high-dose regime of Proleukin because of its short half-life, and the potential to expand Tregs, has raised concerns about the use of this therapy. MDNA11 is a new IL-2R agonist designed to address the limitations associated with IL-2 therapy (86, 87). This has been made possible by implementing two crucial modifications: first, the selectivity of the receptors has been altered in favor of anti-cancer immune cells, which has led to a significant improvement in therapeutic effectiveness, and second, the immunotherapeutic has been fused with albumin to prolong its half-life, eliminating the need for a high dosage of administration (87). MDNA11 exhibits an increased attraction to CD122 (IL-2 receptor on NK and naïve CD8 T cells), but does not bind to CD25 on Treg cells. This leads to a restriction of Treg stimulation, while simultaneously prompting an amplified activation of NK and naïve CD8 T cells when compared to Proleukin. In *in vivo* models, MDNA11 effectively restricted tumor growth when used alone, and when combined with inhibitors of immune checkpoints, it resulted in tumor eradication with a once-weekly dosing schedule (87).

### 4.2. Metabolic reprogramming in Treg cells

Tumor cells are characterized by dysregulated metabolism that disrupts the metabolism of infiltrating immune cells, resulting in decreased glucose, hypoxic and acidic TME. Improving productive antitumor immune responses by disrupting the metabolic programs required to support the tumor-killing functions of infiltrating effector T cells while minimizing the ability of Treg cells to suppress effector T cells in the TME (88). Many molecular signaling pathways and/or molecules, including AKT–MTOR signaling, TLR signaling, HIF1A, MYC, and FOXP3, have been shown to directly affect metabolic programming and development of Tregs.

#### 4.2.1. Glucose uptake

Glucose is needed for effector T cells to function and kill cancer cells. Similarly, cancer cells use glucose and compete with T cells for it, effectively reducing the amount of available glucose and, consequently, reducing the anti-cancer response (89). However, Tregs are less dependent on glucose uptake due to their master transcriptional regulator FOXP3, which shifts their metabolism from aerobic glycolysis to mitochondrial pathways, allowing Tregs to thrive in TME (90). In addition, in human FOXP3^+^ Tregs, FOXP3 inhibits AKT phosphorylation, which in turn prevents Glut1 (the main glucose transporter of Tregs) from being expressed on the cell surface. The most direct method for metabolic reprogramming of Tregs is the PI3K-AKT–MTOR pathway, which is essential in the control of various metabolic pathways, including glucose metabolism (91, 92). An important signal that appears to abolish the Treg suppressive phenotype is the activation of the PI3K → AKT → MTOR axis. In effector T cells, this axis is crucial for inducing proliferation and achieving effector function. However, in Tregs, this pathway can have a profound negative effect on suppressive activity (91). Low glucose levels in the TME promote Treg cell suppressive activity, as downstream activation of the PI3K → AKT → MTOR signaling pathway renders Tregs unstable. Thus, pharmacological intervention to enable AKT activation increases glucose uptake and glycolysis *via* the upregulation of Glut1 and subsequent destabilization of Tregs. PTEN (phosphatase and tensin homolog) is the main negative regulator of PI3K. PTEN catalyzes the dephosphorylation of the inositol ring 3′-phosphate in phosphatidylinositol-3-phosphate)PtdIns3P(and reverses the PtdIns3K reaction (93). Mice that carried a particular PTEN Treg deletion exhibited a substantial decrease in tumor growth and even experienced rapid regression of tumors (94). In addition, PTEN deficiency in Tregs increases FOXP3 instability, which is likely the result of increased glycolytic metabolism and decreased oxidative phosphorylation (OXPHOS) (95). The effective operation of Tregs depends on the OXPHOS pathway. Studies have shown that deleting key regulators of OXPHOS, namely Peroxisome proliferator-activated receptor gamma coactivator 1-alpha (PGC-1α) or NAD-dependent deacetylase sirtuin-3 can disrupt Treg-dependent suppressive function and impair their survival (96). Histone deacetylase-9 (Hdac9) inhibits myocyte enhancer factor 2 (MEF2), which induces the expression of genes essential to OXPHOS. Interestingly, deleting Hdac9 has been found to increase Treg suppressive function (96). PTEN inhibitor drugs are under active investigation for their immunostimulatory impacts (94). VHL (von Hippel–Lindau tumor suppressor) could be an effective target for the activation of the PI3K → AKT → MTOR pathway. Lee et al. demonstrated that Treg cells deficient in VHL exhibit elevated expression of HIF1-α (hypoxia inducible factor 1 subunit alpha). The family of prolyl hydroxylase domain-containing proteins, namely PHD1, PHD2, and PHD3, play a crucial role in driving Treg programming in metastatic niches. This is achieved through the catalyzation of post-translational hydroxylation of HIF1α, which leads to its degradation (97). The targeted deletion of the HIF1-α E3 ubiquitin ligase VHL in Tregs results in elevated levels of HIF1-α (98). This increase in HIF1-α level impairs Treg stability by upregulating the transcription of glycolytic enzymes and directly binding to FOXP3 and inducing its degradation (98). Moreover, HIF1-α directly binds to the *Ifng* promoter in Tregs, ultimately leading to their conversion into Th1-like cells and the impairment of their suppressive function (99). Basu et al. demonstrated that SC79, a small molecular activator of AKT, is able to overcome the effects of FOXP3-mediated AKT repression, leading to significant upregulation of Glut1 in both primary Tregs and conventional T cells (92).

#### 4.2.2. Lipid metabolism

The elevation in lipid oxidation implies that this metabolic route may serve as a crucial source of energy for Treg cells, which is in line with the recent findings that these cells can maintain their viability even under conditions of glucose deprivation or exposure to 2-deoxyglucose, a glycolysis inhibitor (100). However, a comprehensive understanding of how lipids regulate the development and function of Tregs is still lacking. The two most highly activated processes in activated Tregs obtained from human liver cancer are glycolysis and lipid biosynthesis. The utilization of various metabolic pathways may serve as an immune escape mechanism that grants a selective advantage to Tregs in the TME (101, 102). As a result, disrupting lipid metabolism could potentially serve as a therapeutic target for cancer therapy. Several studies have revealed that the genes responsible for lipid metabolism, including CD36, a fatty acid(FA) transporter, were found to be upregulated in TI-Treg cells, playing a crucial role in their ability to suppress antitumor CD8+ T cell responses. Notably, mice with Treg cell-specific loss of CD36 were found to exhibit a reduced count of Treg cells in their tumors, but higher numbers of TI-CD8 + T cells that produced IFN-γ and TNF (tumor necrosis factor) (103). In mice with tumors deficient in Cd36, administration of monoclonal antibodies to PD-1 was found to limit tumor progression and increase survival compared with wild-type mice. This suggests that the antitumor effect of CD36 blockade can be enhanced by reinvigorating exhausted T cells using ICIs. These results indicate that targeting CD36 in Treg cells has the potential to reprogram the TME to immunostimulatory conditions, which may therapeutically complement the effect of PD-1 blockade to combat T cell exhaustion (104). Lim et al. showed that inhibition of FAS (Fatty acid synthase) and SREBF (sterol regulatory element binding transcription factor)-dependent metabolic signaling in Treg cells induces potent antitumor immune responses without autoimmune toxicity (105). In an *in vitro* model of Treg proliferation, treatment with 5-tetradecyloxy-2-furoic acid (TOFA), an ACC (acetyl-CoA carboxylase) inhibitor, −a crucial enzyme in the FAS cascade - significantly reduced Treg proliferation in a dose-dependent manner. TOFA treatment specifically abolished FA accumulation in proliferating cells, demonstrating the importance of FAS in both Treg expansion and lipid pool formation (102). Signaling between CD70 and CD27 was identified as the only significant contact interaction between CD4+ naïve T cells, Tregs, and nasopharyngeal carcinoma (NPC) cells in the TME (106). This interaction strengthens a lipid signaling network in Treg cells that involves mitochondrial integrity, cholesterol homeostasis, and FA metabolism. Knockout of CD70 has been shown to inhibit Treg-mediated suppression, restoring CD8+ T cell immunity. CD70 blockade may act synergistically with anti-PD -1 treatment to reinvigorate T cell immunity against NPC. Anti-CD70+ anti-PD-1 therapy has been studied in preclinical animal models and showed enhanced tumor-killing efficacy (106).

#### 4.2.3. Amino acid metabolism

The metabolism of amino acids provides support for the synthesis of proteins and nucleotides crucial for the rapid proliferation of Treg cells. Nevertheless, the signaling of amino acids in Treg cells and their significance *in vivo* are presently obscure. During the proliferation and activation of Treg cells, a number of major amino acid transporters are expressed, including LAT1, LAT4, CAT-1, SLC3A2, and SLC7A11 (107, 108). For example, the amino acid transporter SLC3A2 is required for maintaining the proliferative state of Treg cells and their suppressive activity by transporting branched-chain amino acids (BCAAs), including leucine, isoleucine, and valine (109, 110). Knockdown of *Slc3a2* in mice results in impaired *in vivo* replication and reduced numbers of Treg cells (110). In addition to BCAAs, arginine, glutamine, serine, tryptophan, glutathione, and glutamate modulate Treg production and function. Tryptophan catabolism is mediated primarily by the rate-limiting enzyme IDO, and there is increasing evidence that IDO-induced metabolites of tryptophan, including kynurenine and 3-hydroxyanthranilic acid (3-HAA), promote the proliferation of Tregs (111). Recently, a correlation between the IDO signaling pathway and Treg biology has been showed in several stages. The first link is attributed to the ability of IDO -expressing dendritic cells (DCs) to drive the differentiation of naïve CD4 (+) T cells toward a Treg phenotype. The second link is the ability of IDO-expressing DCs to activate Tregs and significantly enhance target cell suppression. Finally, the third link is attributed to the ability of IDO to deter the inflammation-induced transformation of Tregs into T helper-like cells. These findings emphasize IDO’s potential as an appealing target for therapeutic intervention, given its involvement in stabilizing and strengthening the suppressive phenotype and deterring the reprogramming of Tregs into T helper-like cells (112). Numerous preclinical and clinical trials have been carried out to examine the effectiveness of IDO1 inhibitors, with the most studied ones being NLG-8189, Epacadostat (INCB024360), NLG-919 (GDC-0919), and Linrodostat (113). The signaling of MTORC1 (mechanistic target of rapamycin complex 1) kinase plays a crucial role in the Treg cell responses to amino acids, specifically arginine, and leucine (108). The activation of MTORC1 is amino acid-dependent and required the activation of GTPases such as RRAG (RRAGA or RRAGB) and RHEB (RHEB1 or RHEB2). The knockdown of these GTPases causes a decline in Treg cell accumulation and suppressive function and is associated with the emergence of deadly autoimmunity (108). The identification of RRAG and RHEB as downstream targets of amino acid signaling implies the potential therapeutic target of these proteins to regulate Treg cell responses in autoimmune diseases and cancer.

#### 4.2.4. Autophagy

**Figure 1:**
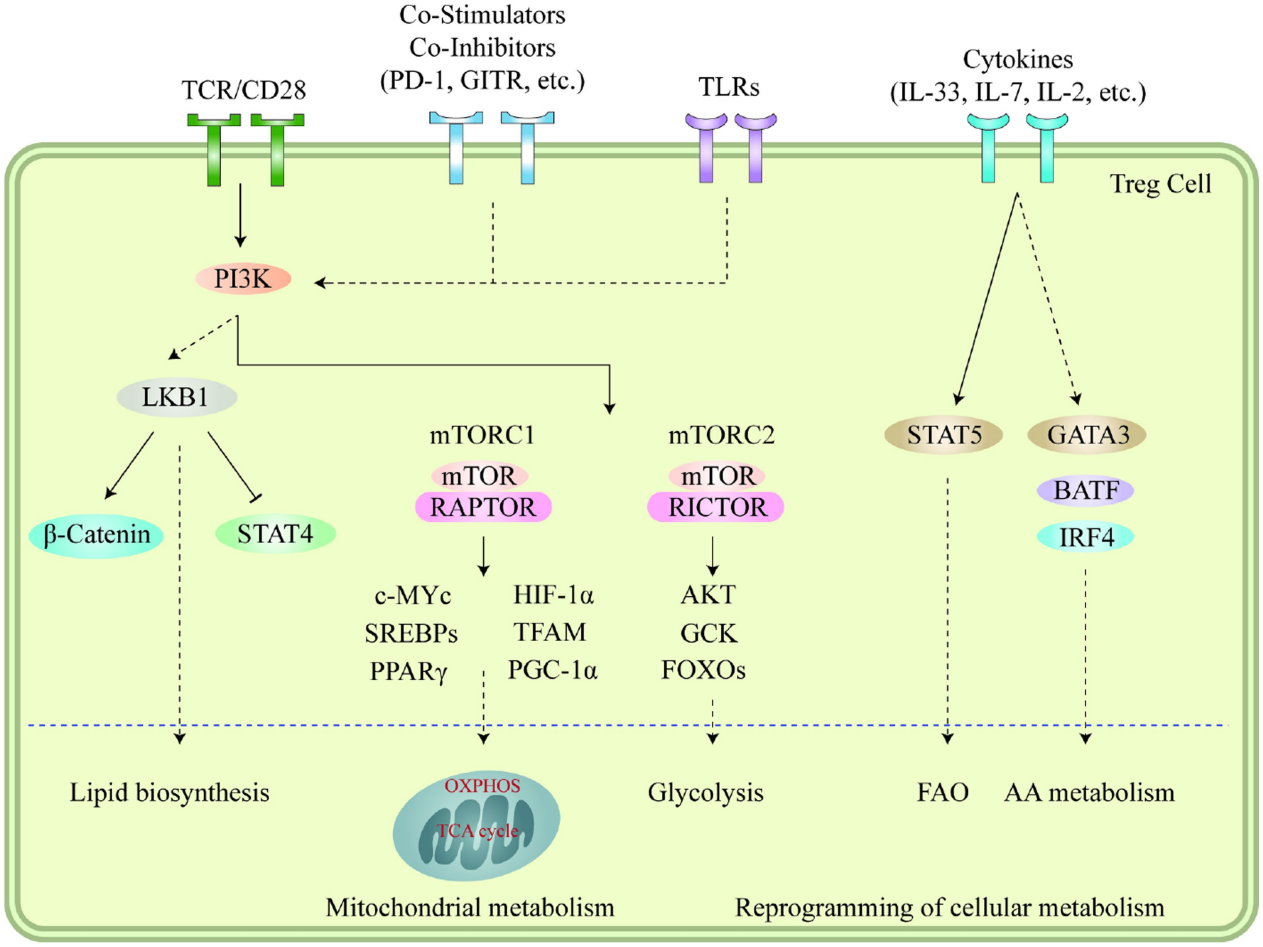
Immune signaling pathways play a vital role in regulating the metabolism and adaptation of Treg cells to non-lymphoid tissues. Stimuli like microenvironmental antigens, co-stimulators, toll-like receptors, and cytokines cause the PI3K signaling pathways to respond to TCR and other signals which then activate mTOR complexes, subsequently leading to the reprogramming of cellular metabolism through several transcription factors. LKB1 signaling is further fueled by TCR and co-stimulation, which in turn restricts the function of STAT4 and stabilizes the b-catenin content, thus enabling metabolic reprogramming. This further leads to the upregulation of certain genes related to metabolic processes, including the biosynthesis of lipids, mitochondrial metabolism, glycolysis, fatty acid oxidation, and amino acid metabolism. Additionally, cytokines present in different tissues also sustain the accumulation and proper functioning of Treg cells through the mediation of various transcription factors. Moreover, the accumulation of VAT-Treg cells is dependent upon signaling from TCR, FOXP3, and IL-33, while IL-7 is necessary for the homeostasis and proper functioning of skin-Tregs. This figure adapted from Yang (118).

Autophagy and lysosomal activities hold critical significance in governing the metabolic efficacy of Treg cells and their cell lineage stability in the TME. Suppression of ATG7 (autophagy-related 7) or ATG5 (autophagy-related 5) in Treg cells results in elevated apoptosis rates and reduced lineage stability (114). Furthermore, it should be noted that the suppressive impact of autophagy on MTORC1 is a contributing factor to the survival and stability of Treg cells. In Treg cells that are deficient in autophagy, the expression of MTORC1 is dysregulated, which in turn promotes the expression of c-Myc and glycolysis metabolic pathway (115). The stability impairment of autophagy-deficient Treg cells is partially restored by pharmacologically inhibiting MTORC1, MYC, or glycolytic activities (114). Conversely, lysosomal knockout of *Traf3ip3*/*T3jam* (TRAF3 interacting protein 3) in mice impairs Treg cell suppressive functions and loss of signature gene *Foxp3* expression, leading to the development of inflammatory diseases, stronger antitumor T cell responses, and a significant reduction in tumor size (116). TRAF3IP3 actively suppresses MTORC1 signaling at the lysosome and limits glycolytic metabolism, thereby preserving Treg cell identity and function (116). The Foxp3CreAtg7fl/fl mouse model was used to illustrate the effects of Treg-restricted autophagy deficiency on MC38 colon adenocarcinoma cells that were inoculated (117). Tumor growth was significantly reduced and the tumor site exhibited a remarkable increase in TI -CD8+ cells, increased IFN-γ expression in effector CD4+ and CD8+ T cells, and a remarkable decrease in Tregs (117). These results demonstrate the critical role that autophagy plays in Treg-mediated suppression of anti-tumor immune responses. Figure 1 illustrates immune signaling pathways involved in the regulation of Treg cell metabolism and adaptation to non-lymphoid tissues.

### 4.3. Treg reprogramming beyond reversal of its immunosuppressive effect

Recent studies have shown that Tregs present in the TME have the ability to not only suppress the anti-tumor immune response but also induce certain properties associated with the tumor, such as proliferation, invasion, and metastasis. This suggests that the reprogramming of Tregs beyond the reversal of their immunosuppressive function could potentially be used to enhance the overall survival of patients by decreasing the possibility of invasion and metastasis (119).

The investigation into the correlation between the direct involvement of Tregs in the metastasis of cancer is currently ongoing and dynamic (120). Previous studies have shown that there is an association between the presence of Tregs and higher metastasis rates in RCC (121), breast cancers (BC) (122), and gastric cancers (GC) (123). TI-Tregs have been shown to stimulate BC cell metastasis through their expression of TNFSF11 (TNF superfamily member 11). TNFSF11 interacts with TNFRSF11A receptor on BC cells, decreasing the expression of SERPINB5 as a metastasis inhibitor and subsequently inducing epithelial-mesenchymal transition (EMT) (124). These results are consistent with the impact of Treg cells on the poor prognosis of human BC and suggest that targeting TNFSF11-TNFRSF11A can be used as a novel therapeutic target in primary breast tumors to curb recurrent metastatic disease (124). Interestingly, in a mouse melanoma model, the combination of antibodies against TNFSF11 and CTLA4 inhibits tumor growth and metastasis, accompanied by increased T-cell effector function due to significantly higher T-cell infiltration into the tumor (125). A fully human monoclonal antibody called denosumab binds to TNFSF11 with great avidity and prevents signaling and interaction between TNFSF11 and TNFRSF11A. Denosumab is widely used to prevent skeletal-related events associated with bone metastases in solid malignancies such as breast and prostate cancer (126).

## 5. Treg-based therapies in autoimmunity

Therapeutic approaches to treat or control autoimmune diseases focus on reviving immune homeostasis and tolerance by promoting, activating, or administering Tregs (127). These therapeutic modalities employ pharmacological agents to inhibit effector T cell function and promote Tregs. Clinicians are increasingly utilizing Treg-mediated suppressive mechanisms as a means of treating autoimmune diseases due to safety guidelines (127). Several Treg-based therapies are now being investigated for the cure and prevention of autoimmune diseases, including polyclonal Tregs, autoantigen-specific TCR Tregs, autoantigen-specific CAR Tregs, and fecal transplantation of specific Treg-promoting bacteria. There are two main sources of Tregs for cell therapy: (1) *in vivo* induction of antigen-specific Tregs by the use of monoclonal antibodies (mAbs), particularly anti-CD3 mAb (teplizumab, and Otelixizumab), and (2) *ex vivo* expanded polyclonal Tregs isolated from peripheral blood and expanded *in vitro* with anti-CD3/CD28 antibody-coated beads and high-dose IL2 (128). In the next section, we have discussed in detail the process of CAR and TCR Treg in the treatment of autoimmunity.

### 5.1. CAR-Treg therapy

CAR is a strategy to generate antigen-specific Tregs in a manner independent of the major histocompatibility complex (MHC) and less dependent on IL2, presenting “off-the-shelf” capability in a translational setting (129). The CAR construct is composed of an extracellular antigen recognition domain that is in a single-chain variable antibody fragment (scFv) form, combined with an extracellular hinge, a transmembrane region, and intracellular signaling domains (130). This particular configuration allows for the transmission of an extracellular signal of the antigen to the Treg cells, which subsequently results in the stimulation of Treg cell activation without the need for interaction with the antigen in the MHC-dependent manner. Based on previous preclinical and clinical results, CAR-Tregs have shown a promising therapeutic potential to treat autoimmune diseases and to induce graft tolerance (131, 132). Two clinical studies are currently underway (NCT04817774 and NCT05234190) using CAR-Tregs to recognize the HLA-A2 molecule on the donated organ and thus induce and maintain immune tolerance to the organ (133, 134).

Type 1 diabetes, commonly referred to as juvenile diabetes, is caused by a breakdown in immunological tolerance toward self-antigens. Extensive research has demonstrated that the absence of Treg cells or their suppressive activity may be a contributing factor toward the lack of self-tolerance among T1D patients. Consequently, utilizing CAR technology to restore the immunological tolerance of Tregs may present a promising approach to combating T1D (135). Tenspoldea et al. produced CAR-Tregs specific to insulin and explored the potential to restore immunological tolerance in T1D using a significant number of CAR-modified Tregs. Despite not being able to prevent diabetes in NOD/Ltj mice, the resulting insulin-specific CAR-Tregs demonstrated a normal Treg phenotype and were long-lasting for diabetic mice. Additionally, they exhibited suppressive properties, as evidenced by their ability to suppress effector T cell proliferation *in vitro* (136). In a separate investigation, Imam et al. conducted a study wherein CAR-Tregs that target beta cells (GAD65 B-cell epitopes) were developed and subsequently employed for therapeutic purposes in a mouse model that closely resembling human T1D (137). The outcomes of this study demonstrated that GAD65-CAR-Tregs successfully colonized the pancreatic islets just 24 h after infusion. Furthermore, the population of Tregs was markedly increased in the pancreas of treated mice in comparison to their untreated counterparts. The glucose tolerance tests (GTT) have demonstrated that the mice subjected to CAR-Tregs treatment exhibited considerably diminished blood glucose levels in comparison to the control group of mice. These findings clearly indicate that the application of CAR engineering in the production of robust, operational, and persistent beta cell-specific CAR-Tregs can serve as a viable therapeutic intervention for treating T1D in humans (137). T1D vaccine candidates can also promote the induction of human FOXP3^+^ Tregs in humanized mice. Serr et al. provided evidence for human autoantigen-specific FOXP3^+^ Treg induction *in vivo* using humanized NSG-HLA-DQ8 transgenic mice (138). They identified HLA-DQ8-restricted insulin-specific CD4^+^ T cell responses and demonstrated efficient insulin-specific FOXP3^+^ Treg induction following subimmunogenic application of insulin mimetopes in a human immune system *in vivo*. They demonstrated that high frequencies of insulin mimetope-specific Tregs are associated with a significant delay in the progression of T1D in children. This supports the consideration of inducing insulin-specific FOXP3^+^ Tregs to delay or even prevent T1D in humans (138).

Multiple sclerosis (MS), a disease of the central nervous system, is characterized by autoimmune demyelination and neurodegeneration and leads to lifelong disability. Its pathogenesis is associated with T cells, making the use of Treg cell therapy a potential treatment option. The rationale behind this approach is that patients with MS have been found to have less functional (although not necessarily less frequent) Tregs (139). In a mouse model of MS, the adoptive transfer of CAR-Tregs has been shown to be effective and resulted in a reduction in disease symptoms. Fransson et al. have reported that CAR-engineered Tregs targeting MOG (myelin oligodendrocyte glycoprotein) in the CNS can suppress the mouse model of experimental autoimmune encephalomyelitis(EAE) when administered intranasally, leading to a reduction in disease symptoms (140).

It is worth mention that, in patients diagnosed with MS, the application of oleic acid *in vitro* has been observed to partially restore the suppressive function of Tregs (141). This discovery suggests that an oleic acid-enriched diet may be considered as an adjuvant therapy for MS patients. Moreover, the binding of oleic acid to the cell surface of Tregs has been found to increase the expression of genes required for fatty acid oxidation (FAO)-driven OXPHOS pathway. This, in turn, reinforces the expression of FOXP3 and enhances the suppressor function of Treg cells (141). The signaling of Prostaglandin I2 (PGI2)-prostacyclin has been found to provide support to the suppressive function of Tregs. In autoimmune diseases, the administration of PGI2 could prove to be an efficacious therapeutic agent for the improvement of Treg function (142). It has been observed that mice lacking the PGI2 receptor exhibit a lower degree of suppressive effect when it comes to allergic airway inflammatory responses, compared to mice with intact PGI2 signaling (142).

### 5.2. TCR Treg therapy

Compared with CAR-Treg therapy, T cell receptor-modified Treg (TCR-Treg) therapy has no restrictions on the expression of antigens on the surface of target cells, which is a significant obstacle to the use of CAR -Treg in the clinic (143). Moreover, TCR-Tregs require only interaction with a peptide–MHC for activation, which differs from CAR -Treg, which requires the presence of more than 100 target self-antigens on the target cell for successful recognition by CAR and subsequent Treg stimulation (144). However, mismatched hybridization of exogenous and endogenous MHC receptor chains may limit their application In NOD mice, which spontaneously exhibit a disease that bears resemblance to human T1D, and in EAE, a model that mimics multiple sclerosis, TCR-engineered Tregs that are targeted toward MBP (myelin basic protein) effectively suppress the development and the progression of the disease. Recent research has shown that TCR-engineered Tregs targeting MBP can effectively suppress MBP-specific T effector cells, as well as T cells with other specificities after Treg activation *via* the TCR. MBP-reactive Treg cells improve EAE recovery in recipient mice when used directly from donor mice (genetically modified mice expressing a transgenic MBP-reactive TCR). *In vitro* expanded MBP-reactive Treg cells also prevent disease progression when administered after the onset of clinical EAE (145). *In vitro*, these Tregs have demonstrated an increase in the expression of key markers, such as FOXP3, LRRC32 and IKZF2 (146). It is noteworthy that these TCR-engineered Tregs have shown functional efficacy, even in the presence of TLR-induced solid inflammatory signals. MBP-specific Tregs have successfully mitigated EAE in MOG (myelin oligodendrocyte glycoprotein)-immunized transgenic mice. Further *in vitro* experimentation has revealed that IL-2 secretion by neighboring effector T cells activates MBP-specific Tregs, initiating suppression of T effectors within the local milieu (147). Similarly, TCR-engineered Tregs targeting MOG and neurofilament medium (NF-MT) were utilized to treat C57BL/6 mice with EAE induced by MOG35–55 or PLP178–191. These manipulated Tregs mitigated the clinical symptoms of EAE, and their effectiveness was noted to increase toward the peak of the disease. The TCR-engineered Tregs that were specific for MOG were equally efficacious in reducing EAE induced by an unrelated central nervous system (CNS) antigen, PLP178–191. Hence, this finding suggests that if extrapolated to humans, this approach could be a valuable therapeutic property in the management of MS (148). Another study demonstrated that induced regulatory T cells (iTregs) that have been engineered with TCR and target proteolipid protein139–151/lipophilin peptide can effectively suppress the T cell response to the PLP139–151 peptides in experimental EAE without inducing pan-suppression *in vivo*. These iTregs are generated *in vitro* by stimulating T cells with TGF-β, retinoic acid, and IL-2. These iTregs underwent antigen-driven proliferation and impeded the proliferation and activation of CD4+ T cells specific to PLP139–151 in SJL/B6 F1 mice that have been primed with PLP139–151 (149).

The primary obstacle in utilizing antigen-specific Tregs in clinical practice is the isolation of Tregs with rare specificities from the native polyclonal T cell repertoire. A novel approach to instantly prevent tissue damage caused by previously activated T cells in autoimmune diseases involves converting primary T cells into antigen-specific regulatory cells. Wright et al. transferred FOXP3 and TCR genes to transform conventional CD4^+^ T cells into antigen-specific regulators for the purpose of facilitating adoptive T-cell therapy of arthritis (150). They used OTII-TCR, which targets SERPIN (SERine Proteinase INhibitors) presented by MHC class molecules II. After adoptive transfer into recipient mice, FOXP3 TCR-transduced CD4^+^ T cells gather specifically in the draining lymph nodes of the SERPIN/ovalbumin-loaded knee, resulting in a local decline in the numbers of inflammatory Th17 cells and a substantial drop in arthritic bone destruction (150). This strategy provides the opportunity to use induced Tregs from CD4^+^ T cells for highly targeted inhibition of tissue damage without systemic immunosuppression. Moreover, opens the possibility of targeting Tregs with tissue-specific antigens to treat autoimmune tissue damage without knowing the autoantigen responsible for the disease.

### 5.3. Metabolic and epigenetic reprogramming

The dysregulation of T helper 17 cells (Th17) and Treg cells has been identified as a prominent etiological factor in various autoimmune diseases (151). It has been observed that methylidenesuccinic acid (itaconate), an endogenous metabolite that is associated with inflammation, can effectively reprogram metabolic and epigenetic processes (152, 153). In Th17- and Treg-polarizing T cells, itaconate can suppress the metabolic pathway that is responsible for glycolysis and electron transport-linked phosphorylation. This suppression leads to inhibition of Th17 cell differentiation and promotion of Treg cell differentiation. In both Th17 and Treg cells, itaconate has been observed to result in a reduction in the concentrations of SAM (S-adenosyl-L-methionine) to the levels of SAH (S-adenosylhomocysteine) and 2-hydroxyglutaric acid (2-HG), respectively (153). These metabolic alterations have been linked to changes in genome accessibility and gene expression during differentiation of Th17 and Treg cells, including decreased binding of RAR-related orphan receptor gamma (RORγt), a member of the nuclear receptor family of transcription factors (154), at the CTLA8 (cytotoxic T-lymphocyte-associated protein 8)/IL-17A promoter (155). The transfer of itaconate-treated Th17-polarizing T cells significantly treated experimental EAE, suggesting that itaconate, as a crucial metabolic regulator for Th17/Treg cell balance, might serve as a therapeutic agent for autoimmune diseases (153).

## 6. Key challenges and future steps

Researchers worldwide have extensively investigated the potential of Treg therapy. The preclinical studies conducted on animal models have yielded valuable insights into the safety, efficacy, and feasibility of Treg therapy. Subsequently, several phase I clinical trials and a few phase II trials have been initiated, indicating that Treg therapy is well-tolerated and holds some promise of efficacy (156). Despite the positive results of Treg therapy in treating autoimmune diseases, several obstacles still need to be addressed. In an experimental context, adoptive cell transfer (ACT) of Treg cells is being utilized to treat autoimmune diseases such as systemic lupus erythematosus and RA. Nonetheless, the isolation of functional Treg cells in higher quantity for Treg cell-based therapies has become challenging (150, 157). Furthermore, the requirement for *ex vivo* manipulations that are considerably expensive and complex, coupled with the risk of contamination with nonTreg cells, serves to limit the effectiveness of their utilization. A novel approach to addressing these challenges involves the generation of functional Treg cells from induced pluripotent stem (iPS) cells (157). Through transduction of the Foxp3 gene and stimulation with a NOTCH ligand *in vitro*, the differentiation of iPS cells into Treg cells is initiated. These Treg cells, which are derived from iPS cells, have the potential to be therapeutic, as they are capable of secreting TGFB and IL10, and interfering with other immune cell activities. In mouse models, Treg cells that are derived from iPS cells have been demonstrated to suppress arthritis, as well as recipient autoimmunity in allogeneic (MHC incompatible) status (157).

Several pre-clinical studies have exhibited the effectiveness of TI-Treg reprogramming because of its ability to reduce Treg immunosuppression in tumors. In fact, it can even reverse it by converting TI-Tregs into pro-inflammatory cells that stimulate the immune response within tumors. However, targeting TI-Tregs for their unique properties in the cancers described here may have negative effects by blocking the function of beneficial immune cells that attack cancer. For example, the development of CD28 blockers may allow for more targeted cancer therapy by interrupting the activation and development of Treg cells. However, it should be noted that CD28 is also crucial for T-cell activation and facilitates maximal glucose uptake by promoting Glut1 subsequent to TCR-induced stimulation (158). Furthermore, it has been shown that the co-stimulation of CD28 has a significant effect on the effectiveness of anti-PD1 therapy (159). The inhibition of CD28, which impairs the activity of effector immune cells that infiltrate the TME, thus counteracts the numerous advantages of Treg reprogramming. Similarly, inhibiting CD25 could potentially impede the anti-neoplastic functions of both NK cells and T effector cells. The expression of CD25 is observed on activated NK cells and is indispensable for their proliferation as well as the production of lytic agents including PRF1 (perforin 1) and GZMB (granzyme B) (160, 161). Therefore, further investigation is necessary to precisely target these pharmaceutical substances toward TI-Tregs, without eliciting negative impacts on immune cells that fight against cancerous cells.

## Author contributions

HH and AR involved in conception, design, statistical analysis and drafting of the manuscript. FR, NE, HR, and DJK contributed in involved in the conception, interpretation of data, drafting and critically revised ms. This author approved the final version of ms. Also, they agree to be accountable for all aspects of the work in ensuring that questions related to the accuracy or integrity of any part of the work are appropriately investigated and resolved. All authors contributed to the article and approved the submitted version.

## Figures and Tables

**Table 1:** 

Tregs	regulatory T cells
CAR	chimeric antigen receptor
IDO	indolamine-2,3-dioxygenase
Th	T helper
CAF	cancer-associated fibroblasts
TAM	tumor-associated macrophages
PD-L1	Programmed death-ligand 1
T1D	type 1 diabetes
MS	multiple sclerosis
CTLA4	cytotoxic T-lymphocyte associated protein 4
GITR	Glucocorticoid-induced TNFR-related protein
NRP1	neuropilin 1
NOD	nonobese diabetic
CBM	CARMA1/Bcl10/Malt1
RA	rheumatoid arthritis
TME	tumor microenvironment
ICIs	immune checkpoint inhibitors
GITRL	Glucocorticoid-induced TNF-related ligand
IL2RA	interleukin 2 receptor subunit alpha
STAT5	signal transducer and activator of transcription 5
PTEN	phosphatase and tensin homolog
OXPHOS	oxidative phosphorylation
VHL	von Hippel–Lindau
HIF1-α	hypoxia inducible factor 1 subunit alpha
DCA	dichloroacetate
PDHK	pyruvate dehydrogenase kinase
2-DG	2-deoxyglucose
FAS	fatty acid synthesis
SREBF	sterol regulatory element binding transcription factor
TOFA	5-tetradecyloxy-2-furoic acid
ACC	acetyl-CoA carboxylase
CPT1	carnitine palmitoyltransferase 1
FAO	fatty-acid β-oxidation
BCAAs	branched-chain amino acids
3-HAA	3-hydroxyanthranilic acid
Arg	arginine
ATG7	autophagy related 7
*Traf3ip3*	TRAF3 interacting protein 3
RCC	renal cell carcinomas
BC	breast cancers
GC	gastric cancers
EMT	epithelial-mesenchymal transition
TNFSF11/RANKL	TNF superfamily member 11
MHC	major histocompatibility complex
scf	single-chain variable antibody fragment
GTT	glucose tolerance tests
MOG	myelin oligodendrocyte glycoprotein
EAE	encephalomyelitis
MBP	myelin basic protein
SERPIN	serpin family B
ACT	adoptive cell transfer
iPS	induced pluripotent stem
EZH2	enhancer of zeste 2 polycomb repressive complex 2 subunit
